# Demoralization syndrome in elderly patients with advanced lung cancer: a multi-center study in North China

**DOI:** 10.3389/fmed.2025.1629264

**Published:** 2025-07-10

**Authors:** Ya Li, Wei Yang, Weiwei Feng, Lingyuan Zhang, Yikun Yao, Kai Yan, Hong Zhang, Xiaochao Wan

**Affiliations:** ^1^2nd Department of Respiratory and Critical Care Medicine, Baoding No. 1 Central Hospital, Baoding, Hebei, China; ^2^Department of Nephrology, Baoding No. 1 Central Hospital, Baoding, Hebei, China; ^3^Department of Psychiatry and Psychology, Army Hospital of the 82nd Group Army, Baoding, Hebei, China

**Keywords:** demoralization syndrome, lung cancer, living alone, social support, self-management

## Abstract

**Objective:**

This observational study sought to explore the prevalence and determinants of demoralization syndrome (DS) among elderly patients in North China diagnosed with advanced lung cancer (ALC), with the primary goal of mitigating their psychological distress.

**Methods:**

A convenience sampling approach was employed to recruit 420 ALC patients aged 60 years or older from four tertiary hospitals in North China. Data collection was carried out using structured questionnaires, which included the Chinese adaptation of the Demoralization Syndrome Scale and Perceptions of Social Support Questionnaire. Statistical analyses were performed using SPSS 26.0 software to derive meaningful insights.

**Results:**

Univariate analysis identified several significant factors influencing DS levels, including gender, smoking, average monthly household income, living alone, stage of cancer, social support, and self-management abilities. The mean DS score was notably elevated, with 32.8% of participants scoring within the high DS range. Multivariable linear regression analysis further established gender, average monthly household income, stage of cancer, social support, and self-management abilities as independent predictors of DS severity.

**Conclusion:**

The findings highlight the critical importance of addressing socioeconomic factors such as household income, strengthening social support networks, improving self-management skills, and implementing targeted interventions for female patients to reduce DS levels and alleviate psychological distress in elderly ALC patients, particularly those with stage IV cancer. These insights provide valuable guidance for healthcare professionals aiming to enhance the overall well-being of this high-risk population.

## Introduction

Advanced lung cancer (ALC) represents a significant global health burden, characterized by high mortality rates and substantial psychological distress among patients. Despite advancements in oncology, the prognosis for ALC remains poor, particularly in elderly patients who often face additional challenges such as comorbidities, reduced physical function, and social isolation (1). The psychological impact of ALC is profound, with many patients experiencing demoralization syndrome (DS), a condition marked by feelings of helplessness, hopelessness, and a loss of meaning in life (2).

DS is increasingly recognized as a critical psychological state in patients with chronic and life-threatening illnesses. It is distinct from clinical depression but shares overlapping symptoms, including low mood, anhedonia, and social withdrawal. DS is particularly prevalent among cancer patients, with studies indicating that up to 59.1% of patients with chronic diseases experience this syndrome (3). The consequences of DS are severe, leading to diminished quality of life, increased family burden, and, in extreme cases, suicidal ideation.

The elderly population is particularly susceptible to the adverse effects of cancer due to the natural decline in physiological resilience and the increased likelihood of comorbid conditions. Aging is associated with a higher prevalence of chronic diseases such as cardiovascular disease, diabetes, and osteoporosis, which can complicate cancer treatment and exacerbate psychological distress (4). Additionally, elderly patients often face social isolation, loss of independence, and financial difficulties, all of which can contribute to the development of DS.

Given the high prevalence of DS in cancer patients and its detrimental effects, understanding the factors that contribute to DS in elderly ALC patients is essential. Previous research has highlighted the role of demographic characteristics, psychological capital, disease cognition, and coping mechanisms in the development of DS (5–7). Notably, among cancer patients, those diagnosed with lung cancer demonstrate the most pronounced levels of demoralization. Their distress levels are significantly higher compared to individuals with hematological, prostate, or breast cancers (8). The phenomenon of demoralization among cancer patients in China has become a significant area of study, particularly concerning individuals with mixed cancer types (9). Current findings indicate that China exhibits one of the highest incidence rates of cancer-related moral anomie, though the underlying causes and contributing factors require further investigation (2). Notably, this trend is especially prevalent among elderly patients, suggesting a demographic-specific vulnerability that warrants deeper exploration. However, there is a paucity of studies specifically focusing on elderly ALC patients, a population that is particularly vulnerable to psychological distress due to the combined effects of aging and severe illness. This study aims to fill this gap by investigating the prevalence and determinants of DS in elderly ALC patients. By identifying the key factors associated with DS, this research seeks to inform targeted interventions that can alleviate psychological distress and improve the overall well-being of this vulnerable population. The findings of this study have the potential to contribute to the growing body of knowledge on the psychological impact of cancer and to guide clinical practice in the care of elderly ALC patients.

## Materials and methods

### Research subjects

**Figure 1:**
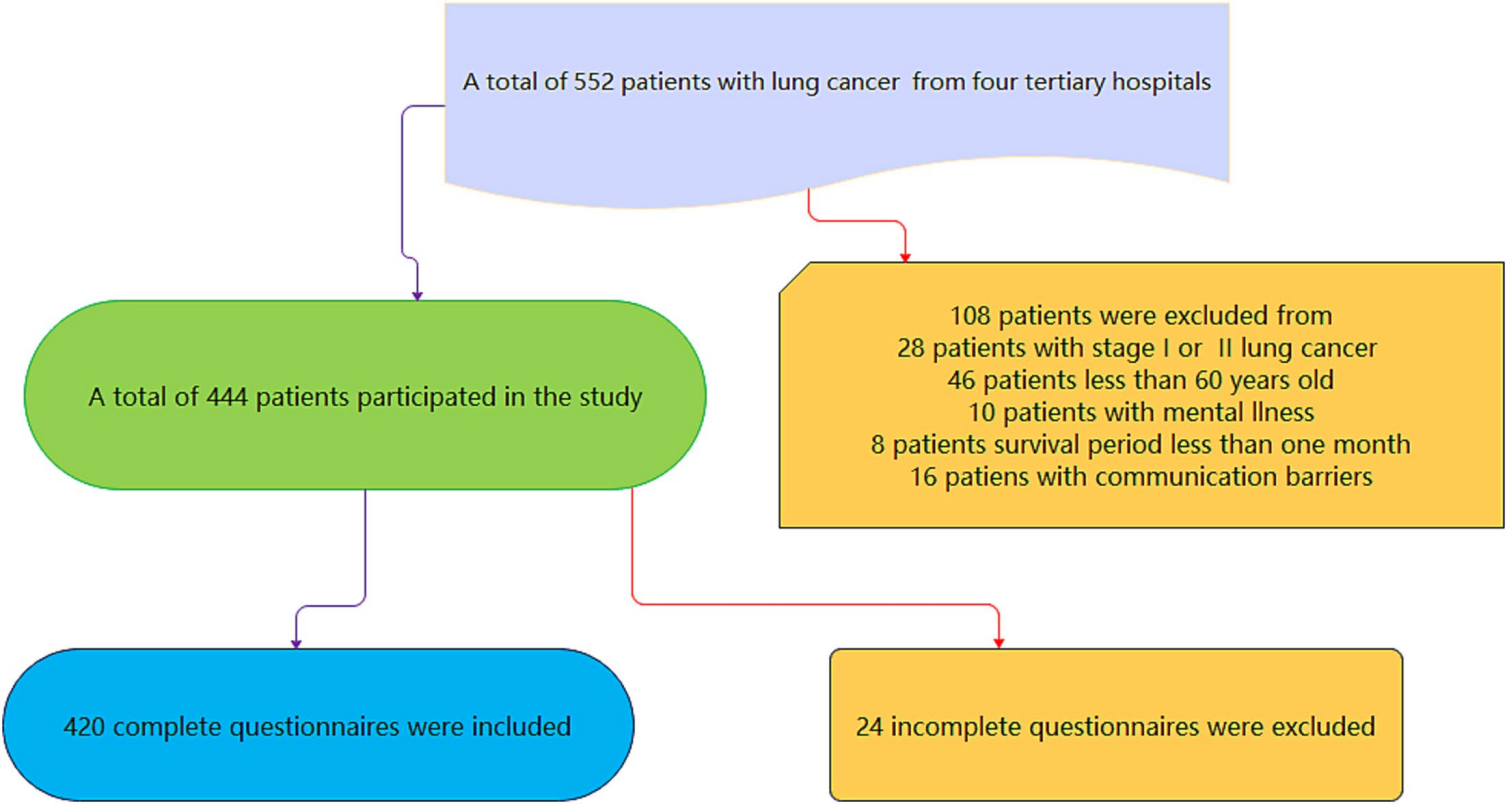
Flowchart of the study.

A convenience sampling approach was used to recruit 420 ALC patients aged 60 years or older from four tertiary hospitals in North China. The study was conducted from June 2024 to December 2024. The inclusion criteria were established as follows: participants had verified diagnosis of lung cancers, with the disease classified as either Stage III or Stage IV. Additionally, individuals were required to be aged 60 years or older and possess the capability to communicate effectively. Exclusion criteria included a history of mental illness or recent major life events such as accidents or bereavement. The sample size was calculated based on the requirement for multiple linear regression analysis, which necessitates a sample size of 5–20 times the number of independent variables. With an estimated 20 independent variables and accounting for a 20% non-response rate, the calculated sample size ranged from 80 to 360 cases. The final sample (*n* = 420) exceeded the upper limit due to higher-than-expected recruitment efficiency across four tertiary hospitals and the large available pool of ALC patients. This oversampling did not alter the planned statistical approach but increased the *post hoc* power to 0.98, particularly enhancing the reliability of subgroup analyses. The study was approved by the Ethics Committee of Baoding No.1 Central hospital (Ethics Approval Number: 2024198), and all recruited patients were informed and willing participants in this study. The flowchart can be seen in Figure 1.

### Research tools

Patient General Information Questionnaire: This questionnaire, designed by the researchers, collected demographic information (age, sex, smoking, drinking, education level, marital status, living alone, healthcare payment methods, average monthly household income, and self-management abilities). Additionally, it captured information regarding the participants’ cancer stage and the extent of social support they received.

Chinese Version of the DS Scale: DS is a psychological disorder marked by significant emotional distress stemming from a sequence of life events (10). This condition often manifests as an individual’s inability to effectively manage stress or a perceived sense of inadequacy in challenging situations. The DS Scale, originally developed by Hong Xiaoqi and colleagues, was adapted from the English version of the Kissane DS Scale. Further cultural modifications were introduced by Liu Peipei and her team to ensure its applicability in assessing the condition of patients experiencing DS. The scale comprises five distinct dimensions, each addressing specific emotional states: meaninglessness (5 items), depression (5 items), unrest (5 items), failure (5 items), and helplessness (4 items). Each item is scored on a scale of 0 to 4, reflecting the severity of the symptom, with a maximum total score of 96. Higher scores on the scale indicate a more severe manifestation of DS. Scores are categorized into three levels: 0 to 30 points (low level), 31 to 60 points (moderate level), and 61 to 96 points (high level). The scale demonstrates strong internal consistency, with a Cronbach’s *α* coefficient of 0.97, indicating high reliability in measuring the construct of DS.

Perceptions of Social Support Questionnaire (PSSQ): The PSSQ, developed by Li et al., measures the degree of social support perceived by individuals (11). It comprises 12 items spread across three dimensions: support from friends, family, and various social circles. Participants’ responses are assessed using a 7-point Likert scale, where a score of 1 indicates “Strongly Disagree” and 7 signifies “Strongly Agree.” This scoring system results in a total possible range of 12 to 84 points. A higher cumulative score reflects a greater level of perceived social support. Specifically, scores are categorized into three distinct levels: a low level of social support is indicated by scores ranging from 12 to 28, a moderate level by scores between 29 and 56, and a high level by scores from 57 to 84. The reliability of the scale is confirmed with a Cronbach’s *α* coefficient of 0.893.

### Data collection methods

Data were collected using a combination of paper-based questionnaires and electronic surveys. Researchers provided uniform training to surveyors, who used standardized guidelines to explain the requirements to participants. For participants with difficulty in writing or reading, surveyors read out each item in a non-leading manner and assisted in filling out the questionnaire. Completed surveys were collected on the spot, checked for missing items, and promptly supplemented to ensure full completion. Upon onsite collection, questionnaires were immediately checked for completeness. For instruments with <5% missing items: mean imputation was applied to continuous variables, while missing categorical data were supplemented using the mode of the respective item. Questionnaires with ≥5% missing items or systematic omissions were excluded as invalid. Ultimately, 24 questionnaires (5.4% of collected samples) were excluded.

### Statistical methods

Statistical analysis was conducted using SPSS 26.0 software. Normality tests were performed on quantitative data, with normally distributed data presented as mean ± standard deviation (x̄ ± s). Non-normally distributed data were represented using median and quartiles. Group comparisons were made using t-tests or one-way analysis of variance. Descriptive statistics such as frequencies and percentages were used for categorical data, with group comparisons analyzed using chi-square tests. Multiple linear regression was employed to analyze factors impacting DS development, with a significance level of *p* < 0.05.

## Results

### Comparison of baseline characteristics for DS in elderly ALC patients

A total of 444 questionnaires were distributed, with 420 valid questionnaires returned, yielding an effective recovery rate of 94.6%. The study included 420 elderly ALC patients, with 59.0% being male and 41.0% female. Among them, 40.0% were between 60 and 70 years old, and 60.0% were over 70 years old (Table 1). This study investigated DS among 420 elderly patients with ALC, identifying several key factors associated with higher demoralization scores. Female patients, smokers, individuals with lower income, those living alone, and patients with stage IV cancer exhibited significantly higher levels of demoralization (all *p* < 0.01, Table 1). Additionally, low social support and poor self-management abilities were strongly correlated with increased demoralization. Notably, participants with stage IV cancer and those living alone reported the highest demoralization scores, underscoring the profound impact of advanced disease and social isolation on mental health. On the other hand, there were no significant differences in age, alcohol consumption, education level, marital status, and healthcare payment methods (*p* > 0.05, Table 1).

**Table 1:** The general data of demoralization syndrome for these elderly patients with advanced lung cancer (*n* = 420).

Variables	*N* (%)	Scores of demoralization syndrome	*P*
Gender, *n* (%)			
Male	248 (59.0)	48.2 ± 11.7	<0.001
Female	176 (41.0)	60.7 ± 12.4	
Age, (Years)			
60 ~ 70	176 (40.0)	59. 4 ± 8.9	0.068
>70	244 (60.0)	61.7 ± 9.1	
Smoking, *n* (%)			
Yes	272 (64.8)	58.2 ± 9.4	<0.01
No	148 (35.2)	43.1 ± 8.8	
Drinking, *n* (%)			
Yes	174 (41.4)	51.2 ± 9.2	0.797
No	246 (58.6)	50.9 ± 8.9	
Education level, *n* (%)			
Middle school or below	88 (20.9)	53.1 ± 8.6	0.165
High school or vocational school	178 (42.4)	49.8 ± 8.3	
College or above	164 (36.7)	52.4 ± 8.5	
Marital status *n* (%)			
Married	352 (83.8)	55.3 ± 8.2	0.782
Unmarred	44 (10.5)	53.9 ± 7.8	
Others	24 (5.7)	54.6 ± 8.5	
Average monthly household income (Yuan), *n* (%)			
<2000	132 (31.4)	61.2 ± 8.9	<0. 001
2000 ~ 5,000	242 (57.6)	52.4 ± 7.2	
>5,000	23 (11.0)	41.6 ± 8.8	
Living Alone, *n* (%)			
Yes	70 (16.7)	62.5 ± 8.5	<0.001
No	350 (83.3)	42.4 ± 7.9	
Healthcare payment methods, *n* (%)			
Rural Cooperative Medical Scheme	196 (46.7)	45. 3 ± 8.6	0.934
Healthcare insurance	146 (34.8)	46.2 ± 8.9	
Others	78 (18.5)	45.7 ± 8.2	
Stage of lung cancer, *n* (%)			
III	118 (28.1)	45.2 ± 8.7	<0.001
IV	302 (71.9)	62.4 ± 8.2	
Social support, *n* (%)			
Low	98 (23.2)	61.4 ± 10.6	<0.001
Moderate	168 (40.0)	52.4 ± 9.8	
High	154 (36.8)	42.7 ± 8.5	
Self-management abilities, *n* (%)			
All	144 (35.7)	42.7 ± 8.6	<0. 001
Partly	246 (58.6)	51.4 ± 7.8	
No	30 (5.7)	63.1 ± 7.4	

### The level of DS in elderly ALC patients

The total score on the DS Scale for these patients was 64.8 ± 8.7 points (Table 2). Among the patients, 90 (21.4%) exhibited low levels of DS, with a mean score of 21.4 ± 7.3. The majority of patients, 192 (45.8%), were classified as having moderate levels, with a mean score of 52.6 ± 6.8 (Table 3). Additionally, 158 patients (32.8%) were identified as having high levels of DS, with a mean score of 69.3 ± 5.4 (Table 3). The data indicate that moderate levels of DS are the most prevalent, affecting nearly half of the patient population, while a significant proportion of patients also experience high levels of the syndrome. Overall, the level of DS was moderately high.

**Table 2:** Score of demoralization in these elderly patients with advanced lung cancer.

Item	Unmeaning	Discouraged	Unrest	Failure	Helplessness	Total score
Scoring range	0 ~ 20	0 ~ 20	0 ~ 20	0 ~ 20	0 ~ 16	0 ~ 96
Scoring (x ± s)	14.4 ± 3.1	11.2 ± 5.2	12.1 ± 3.8	12.7 ± 2.5	12.5 ± 5.3	64.8 ± 8.7

**Table 3:** Classification of demoralization syndrome severity for these patients.

The level of demoralization syndrome status	Frequency (*n* = 420)	Percentage	Score
Low	70	21.4%	21.4 ± 7.3
Moderate	192	45.8%	52.6 ± 6.8
High	138	32.8%	69.3 ± 5.4

### Correlation analysis of DS and each statistically significant variable

We analyzed the correlation between DS and factors such as gender, smoking, average monthly household income, living alone, stage of cancer, social support, and self-management abilities. The results indicated that these factors have significant statistical relevance with DS (*p* < 0.05, Table 4).

**Table 4:** Correlation analysis of demoralization syndrome (Y) and each statistically significant variable (X).

Variables	Demoralization syndrome (Y)						
	Gender	Smoking	Average monthly household income	Living alone	Stage of lung cancer	Social support	Self-management abilities
*r*-value	0.124	0.258	−0.421	0.456	0.257	−0.521	−0.359
*p*-value	<0.05	<0.05	<0.05	<0.05	<0.05	<0.05	<0.05

### Multivariable linear regression analysis of DS in elderly ALC patients

Using the DS score as the dependent variable in the Chinese version, significant variables from the univariate analysis were selected as independent variables. Categorical variables were assigned values as shown in Table 5, while the remaining variables were inputted as their original values. The results of the multivariable linear regression analysis showed that gender, average monthly household income, stage of cancer, social support, and self-management abilities were independent influencing factors of DS (*p* < 0.05, Table 6). Patients with lower average monthly household income, female patients, ALC patients with stage IV, and those with weaker social support and self-management abilities had higher DS scores.

**Table 5:** Categorical variables assignment methodology.

Variables	Values
Sex	Male = 1, Female = 2
Age (Years)	60 ~ 70 = 1, >70 = 2
Smoking	No = 0, Yes = 1
Drinking	No = 0, Yes = 1
Education	Middle school or below = 1, High school or vocational school = 2, College or above = 3
Marital Status	Married = 1, Unmarry = 2, Others = 3
Average monthly household income (Yuan)	<2000 = 1,2000 ~ 5,000 = 2, >5,000 = 3
Living alone	No = 0, Yes = 1
Healthcare payment methods	Rural Cooperative Medical Scheme = 1, Healthcare insurance = 2, Others = 3
Stage of lung caner	III = 1, IV = 2
Social support	Low = 1, Middum = 2, High = 3
Self-management abilities	All = 1, Partly = 2, No = 3

**Table 6:** Multiple linear regression analysis of factors influencing demoralization syndrome in these elderly patients with advanced lung cancer (*n* = 420).

Independent variables	Regression coefficient	Standard error	Standardized regression coefficients	t	*P*
Constant	16.594	2.527	—	12.786	<0.01
Gender	4.581	0.684	0.127	5.864	**0.023**
Smoking	4.627	0.215	0.256	3.121	0.089
Average monthly household income	−2.986	0.427	−0.186	4.357	**0.016**
Living alone	4.257	0.165	0.198	2.126	0.062
Stage of Cancer	1.327	0.493	0.086	4.025	**<0.05**
Social Support	−0.682	0.138	0.037	4.892	**<0.01**
Self-management abilities	−4.863	0.402	−0.126	5.964	**<0.05**

*R*^2^ = 0.542, adjusted *R*^2^ = 0.516, *F* = 56.319, *P* < 0.05. Bold values denote statistically significant independent predictors of demoralization syndrome (*p* < 0.05).

## Discussion

The findings of this study reveal a high prevalence of DS among elderly patients with ALC in North China, with 32.8% of participants scoring in the high DS range. This result is consistent with previous research indicating that DS is a significant psychological burden in patients with chronic and life-threatening illnesses (12). A study showed that DS was prevalent in 30–50% of cancer patients, particularly those with advanced disease, and was strongly associated with reduced quality of life and increased suicidal ideation (13). DS is a common psychological state in patients with terminal illnesses, characterized by feelings of helplessness, hopelessness, and a loss of meaning in life (14). The elevated DS scores in our study population underscore the urgent need for targeted interventions to address the psychological distress associated with ALC, particularly in elderly patients who are already vulnerable due to age-related physical and social challenges.

Our study found that female ALC patients were more susceptible to DS compared to their male counterparts. This finding aligns with existing literature suggesting that women may experience greater psychological distress in the context of chronic illness (15). A research displayed that female cancer patients were more likely to experience DS due to higher levels of anxiety and depression, as well as greater social and emotional burdens (16). The reasons for this gender disparity may include differences in coping mechanisms, social roles, and the tendency for women to express emotional distress more openly (17). These findings suggest that interventions aimed at reducing DS in elderly ALC patients should consider gender-specific approaches, such as providing additional emotional support and coping strategies for female patients.

This study showed that the inverse relationship between household income and DS scores highlighted the impact of socioeconomic factors on psychological well-being. Patients with lower incomes may face additional stressors, such as financial strain and limited access to resources, which can exacerbate feelings of helplessness and hopelessness. This finding is consistent with a study by Wang et al., which found that low socioeconomic status was a significant predictor of DS in burn patients (18). Similarly, Huang et al. reported that financial difficulties were associated with higher levels of demoralization in cancer patients, as they often struggle to afford treatment and manage the economic burden of their illness (19). These findings underscore the importance of addressing socioeconomic disparities in the management of DS, particularly in elderly ALC patients who may already be financially vulnerable due to retirement and limited income sources. Interventions such as financial assistance programs, access to affordable healthcare, and social support services could help alleviate the economic burden and reduce DS levels in this population.

Our research revealed that social support plays a critical role in mitigating DS, particularly among cancer patients. Research indicates that high levels of social support significantly reduce DS levels and improve mental health outcomes (20). Yang et al. demonstrated that social support alleviated psychological distress by providing emotional comfort, practical assistance, and informational resources, enabling patients to better cope with the psychological burden of their illness (21). For instance, emotional support from family and friends can reduce feelings of loneliness and helplessness, while professional support from healthcare teams can enhance patients’ confidence in their treatment. Additionally, Wang et al. found that social support buffers the negative impact of disease severity on DS, with its protective effects being particularly pronounced in advanced cancer patients (22). Conversely, a lack of social support may exacerbate feelings of isolation and hopelessness, leading to heightened DS levels. Therefore, in clinical practice, interventions emphasizing social support—such as establishing support groups or providing psychological counseling—should be prioritized to help patients better navigate the psychological challenges associated with their illness.

This study also found that tumor stage, particularly stage IV lung cancer, was significantly associated with higher levels of DS in elderly patients with ALC, with stage IV cancer patients exhibiting the highest DS scores. These findings are consistent with numerous recent studies, underscoring the critical role of disease severity in influencing mental health outcomes (23). Tumor stage is a key predictor of psychological burden in cancer patients. Those with advanced-stage cancer (e.g., stage IV) often experience more severe physical symptoms, treatment-related side effects, and prognostic uncertainty, all of which collectively exacerbate psychological distress. Research indicates that advanced cancer patients are more prone to anxiety, depression, and demoralization (24). Patients with advanced cancer often face a higher risk of mortality and treatment failure, and this uncertainty can lead to feelings of helplessness and despair, further intensifying DS levels.

Additionally, this study showed that self-management abilities were also found to influence DS levels. Patients with poor self-management abilities may struggle to adhere to treatment regimens and cope with the physical and emotional demands of ALC. These findings are consistent with a study by Allegrante et al., which found that self-management skills were a key factor in maintaining and enhancing the quality of life for patients with chronic diseases (25). Similarly, Markle-Reid et al. reported that improving self-management abilities could enhance the quality of life for elderly patients with chronic illnesses (26). These findings suggest that interventions aimed at improving health literacy and self-management skills, such as patient education programs and self-management training, could play a crucial role in reducing DS levels in this population.

Based on the identified predictors of DS, we propose the following evidence-based interventions: (1) Financial toxicity mitigation: Provide in-hospital financial counseling for patients with monthly incomes below ¥2,000, assisting with insurance claims and enrollment in charitable drug assistance programs (e.g., targeted therapy subsidies), while establishing community-based “Cancer Patient Mutual Aid Funds” to offset out-of-pocket expenses. (2) Gender-specific psychological support: Implement women-only support groups (mean DS scores: 60.7 ± 12.4 in females vs. 48.2 ± 11.7 in males), employing narrative therapy to facilitate emotional expression, coupled with caregiver training in “empathic care” techniques to address patients’ helplessness. (3) Social support enhancement: Forge partnerships between tertiary hospitals and communities to match volunteers with isolated patients, supplemented by a WeChat mini-program digital platform that delivers tiered psychological resources based on PSSQ scores. (4) Self-management skill building: For patients with limited self-management capacity, conduct structured training covering four domains: symptom monitoring, medication adherence, emotional regulation, and advance care planning.

Despite the valuable insights provided by this study, several limitations should be acknowledged. Firstly, the convenience sampling method used may limit the generalizability of the results. The sample size is relatively small and includes only elderly ALC patients from four tertiary hospitals in China, which may not be representative of other populations or healthcare settings. Future studies could use multi-center data with a larger sample size to validate the results. Secondly, this study is a cross-sectional design, which limits our ability to establish causal relationships between the identified factors and DS. Longitudinal studies should be conducted to further investigate the incidence of DS in ALC patients and identify influencing factors, leading to the development and implementation of targeted intervention strategies. Additionally, some factors that may influence DS, such as specific treatment modalities and comorbidities, were not included in this study. Differential anticancer treatments (e.g., chemotherapy, targeted therapy, immunotherapy) may indirectly affect DS through side effects (e.g., fatigue, pain) or efficacy variations. For instance, patients receiving high-intensity chemotherapy may experience exacerbated helplessness due to poor tolerance. While these variables were not collected, our data show significantly higher DS scores in stage IV patients, aligning with their higher palliative treatment exposure and symptom burden. Future studies should systematically document treatment history and Charlson Comorbidity Index to better disentangle DS predictors.

## Conclusion

In conclusion, this study highlights the high prevalence of DS among elderly ALC patients in North China and identifies several key factors influencing DS levels, including gender, average monthly household income, stage of cancer, social support, and self-management abilities. The results underscore the critical need for targeted interventions that address socioeconomic factors, enhance social support systems, and improve self-management skills to reduce DS levels and alleviate psychological distress in elderly ALC patients. These insights offer practical guidance for healthcare professionals striving to improve the overall well-being of this vulnerable population. By implementing interventions that address the identified factors, healthcare providers can help elderly ALC patients cope more effectively with the psychological challenges of their illness, ultimately improving their quality of life and overall prognosis.

## Data Availability

The raw data supporting the conclusions of this article will be made available by the authors, without undue reservation.
